# A Pilot Study on Motion Intention Mapping and Direct Myoelectric Control Method for Prosthetic Knee Based on LSTM Network and Human-Machine Coupling Model

**DOI:** 10.3390/s25247618

**Published:** 2025-12-16

**Authors:** Xiaoming Wang, Yuanhua Li, Xiaoying Xu, Hongliu Yu

**Affiliations:** 1 School of Health Science and Engineering, University of Shanghai for Science and Technology, Shanghai 200093, China; 2 School of Instrument Science and Engineering, Southeast University, Nanjing 210096, China; 3 Shanghai Engineering Research Center of Assistive Devices, Shanghai 200093, China

**Keywords:** prosthetic knee, surface electromyography (sEMG), LSTM neural network, human-machine coupling model, direct myoelectric control

## Abstract

To enhance the adaptability and human-machine coordination of intelligent prosthetic knees, this study proposes a motion intention mapping direct myoelectric control method based on an LSTM network and a human-machine coupling model. Multichannel surface electromyography (sEMG) and knee joint angle data were collected during level-ground walking. Time-domain features were extracted to construct an LSTM prediction model, enabling temporal mapping between muscle activity and joint kinematics. Experimental results show that the LSTM model outperforms traditional neural networks in terms of prediction accuracy and temporal consistency. Furthermore, by integrating the human-machine coupling dynamics model with a hydraulic actuation system, a direct myoelectric control framework for a variable-damping prosthetic knee was established, achieving continuous damping adjustment and smooth gait transition. The results verify the feasibility and effectiveness of the proposed method in human-machine coordinated control.

## 1. Introduction

Intelligent control of lower-limb prostheses is a crucial research focus in the fields of rehabilitation engineering and human-machine integration. As the central pivot of human lower-limb motion, the knee joint plays a decisive role in walking stability, comfort, and energy efficiency. For above-knee amputees, an intelligent prosthetic knee must not only restore motion functionality but also accurately respond to the wearer’s movement intentions during dynamic walking to achieve a natural and coordinated gait [1].

Traditional prosthetic control strategies, such as finite-state machines, damping switching, and trajectory tracking, typically rely on mechanical feedback signals such as ground reaction forces or joint angles for control [2,3,4,5,6,7]. Although these methods can partially achieve gait-phase transitions, they suffer from response delays and poor adaptability to complex and variable gait environments. To achieve better autonomous control of prosthetic limbs, recent research has proposed direct intention control methods, which decode the amputee’s surface electromyography (sEMG) signals and establish a mapping between these bio-signals and prosthetic joint angles or torques, enabling active and voluntary control of the prosthesis [8,9]. Among these, proportional control was one of the earliest methods used for direct myoelectric control of prostheses [10], in which bio-signals and control targets are mapped through simple mathematical relationships. However, this approach often results in inaccurate mappings and limited control precision [11]. To improve control accuracy, some studies have incorporated musculoskeletal models into the control framework [12]. Nevertheless, the forward dynamic computations required by these models are time-consuming and cannot meet the real-time performance requirements of lower-limb prosthesis control.

With the rapid advancement of artificial intelligence, machine learning and deep learning-based direct myoelectric control methods have become research hotspots [13,14]. Coker et al. [15] employed an artificial neural network (ANN) to achieve continuous prediction of knee joint angles from EMG signals. Li et al. [16] and Sun et al. [17] developed hybrid algorithms combining neural networks and least-squares support vector machines to achieve high-accuracy estimation of hip and knee joint angles. These studies demonstrated that AI algorithms can effectively capture the nonlinear relationship between EMG signals and kinematic features, providing new pathways for direct prosthesis control. Prior studies have shown that sEMG characteristics are strongly affected by anatomical and detection-system factors [18], as well as by changes in joint angle during locomotion [19]. These sources of variability pose challenges for achieving reliable EMG-to-kinematics mapping, further motivating the development of more robust intention-decoding models.

Among these, the Long Short-Term Memory (LSTM) neural network, due to its gated structure and ability to model long-term temporal dependencies, has been widely applied in lower-limb motion prediction [20,21]. However, most existing neural network (including LSTM-based) studies focus on discrete motion pattern recognition and classification, followed by trajectory planning and control. How to establish a continuous mapping relationship between EMG signals and kinematic features for smoother and more natural direct myoelectric driving remains an open research question.

To address this challenge, this study proposes a motion intention mapping and direct myoelectric control method for prosthetic knees based on an LSTM neural network. Multi-channel EMG signals and knee joint angle data were collected from healthy subjects during level walking. An LSTM model was constructed to realize nonlinear mapping between EMG signals and kinematic features and to validate its accuracy and stability in continuous gait prediction. On this basis, combined with the authors’ previously developed human-machine coupling model, the predicted knee angle signals from the neural network are converted into control commands for a hydraulic servo valve, establishing a direct myoelectric control system for a variable-damping hydraulic knee prosthesis. This system enables natural flexion-extension motion of the prosthetic knee directly driven by EMG signals, thereby improving adaptability and human-machine coordination.

## 2. Materials and Methods

### 2.1. Experimental Protocol and Data Acquisition

**Figure 1:**
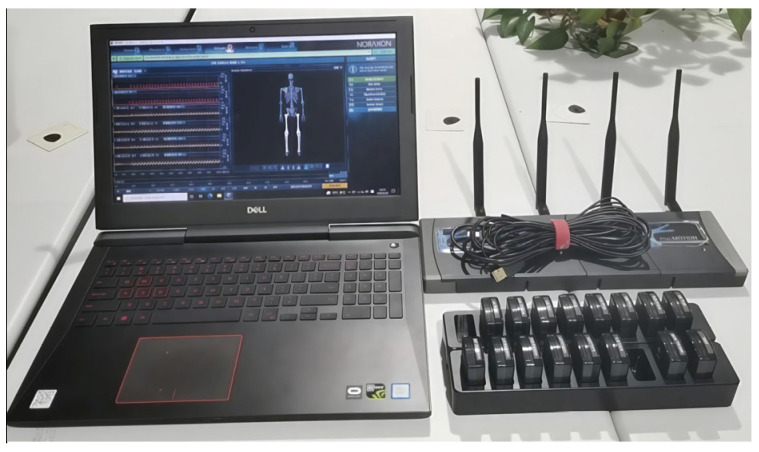
Schematic diagram of the experimental system architecture.

To investigate the mapping relationship between surface electromyography (sEMG) signals and lower-limb joint motion, a multimodal data acquisition platform based on the Noraxon system (Noraxon CO., LTD., Scottsdale, AZ, USA) was established (Figure 1). It comprised three core modules: the Ultium EMG wireless acquisition unit, the MyoMotion 3D motion analysis unit, and the MyoSync synchronization module. The Ultium EMG system recorded multi-channel sEMG signals at 1500 Hz, while the MyoMotion system, using inertial measurement units (IMUs), captured major lower-limb joint angles at 200 Hz. The MyoSync module ensured precise synchronization among all subsystems through a Bluetooth-triggered clock signal, maintaining system latency below 10 ms—sufficient for real-time gait analysis.

The experiments were conducted indoors on a flat surface under controlled environmental conditions. Given that this study represents an initial feasibility investigation of the proposed direct myoelectric control method, experiments were performed using a single healthy subject (aged 26 years, height 178 cm, and weight 70 kg) with no history of lower-limb injury or neurological disorders. It is noted that since EMG availability in amputees varies according to residual-limb musculature, the present study focuses on validating the methodological feasibility in a healthy subject, with future work planned to adapt the approach to amputee-specific muscle conditions. Prior to electrode placement, the target skin areas were cleaned and degreased to reduce impedance. Surface electrodes were attached to the target muscles surrounding the knee joint to capture EMG activity associated with knee flexion-extension. The experimental task consisted of level-ground walking at 0.9–1.2 m/s, each trial lasting 5 min, with five trials in total and a 30-s rest between trials. Before data acquisition, a posture calibration procedure was performed using the MyoMotion system; the participant stood still for 5 s to obtain baseline signals. After data collection, sEMG and kinematic data were synchronized through the MyoSync module. Validation tests confirmed that the timing deviation between the two systems’ trigger pulses was less than 2 ms, ensuring temporal consistency of the recorded data.

**Figure 2:**
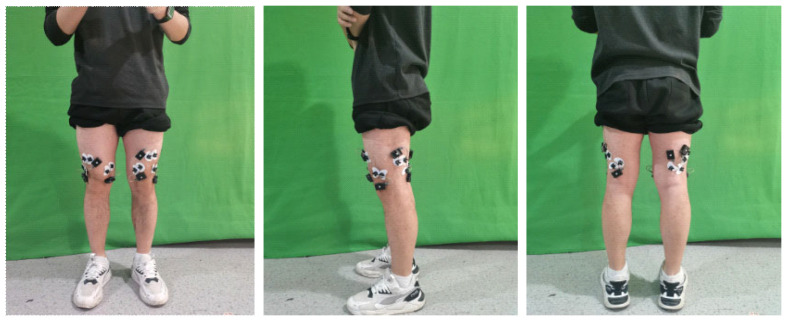
Electrode placement on the target muscle groups.

To accurately capture EMG activity related to knee motion, five major muscle groups were selected for signal acquisition: rectus femoris (RF), vastus lateralis (VL), vastus medialis (VM), biceps femoris (BF), and semitendinosus (ST). The rectus femoris, vastus lateralis, and vastus medialis form the knee extensor group, while the biceps femoris and semitendinosus act as flexor muscles. These muscles were chosen based on their critical functional roles in generating knee flexion-extension torque, which is the principal degree of freedom actuated by the prosthetic knee. Although proximal muscles such as the TFL, gluteus maximus, and adductors contribute to hip stabilization, they were not included in this feasibility-stage study but will be considered in future work to further enrich EMG information. The Noraxon Ultium EMG system, equipped with Ag/AgCl surface electrodes (LT-7, LITU CO., LTD., Shanghai, CHN), was used for high-fidelity signal acquisition. The inter-electrode distance was set to 20 mm to ensure optimal spatial resolution and minimize crosstalk. Electrode placement was determined according to standard anatomical landmarks of each target muscle, as illustrated in Figure 2. The participant provided written informed consent after being informed of the experimental procedures. The study protocol was approved by the Institutional Review Board of Shanghai University of Science and Technology Affiliated Shidong Hospital (Ref. No. IRB-AF63).

### 2.2. Signal Preprocessing and Feature Extraction

#### 2.2.1. Signal Preprocessing

**Figure 3:**
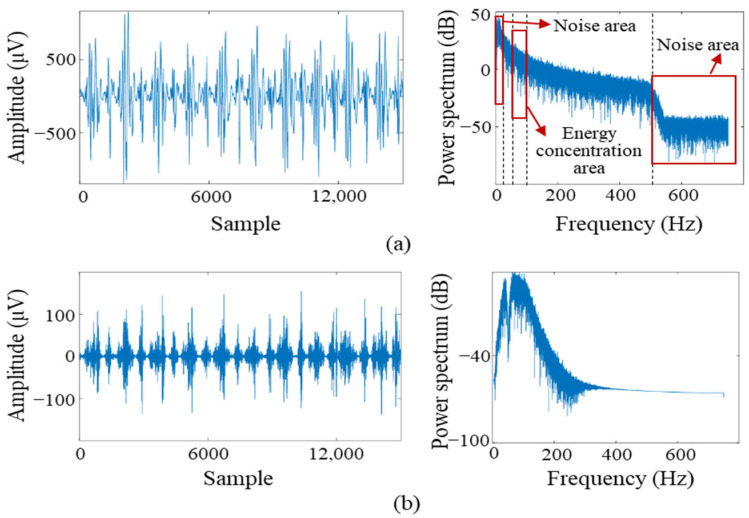
Comparison of sEMG signals from the rectus femoris before and after preprocessing. (**a**) Raw sEMG signal and power spectrum; (**b**) Filtered sEMG signal and power spectrum after applying a Butterworth band-pass (50–100 Hz) and notch filter at 50 Hz.

The sEMG signals are non-stationary bioelectrical signals that are highly susceptible to motion artifacts, ambient noise, and baseline drift. Therefore, effective preprocessing is essential to ensure signal fidelity. As shown in Figure 3a, spectral analysis of the raw rectus femoris EMG signal revealed substantial low-frequency noise below 20 Hz, high-frequency interference above 500 Hz, and a distinct 50 Hz power-line component. Surface EMG signals generally exhibit energy across a broad 20–450 Hz bandwidth, with the dominant and most stable spectral components during level walking typically concentrated around 50–100 Hz [22]. In this feasibility-stage study, the filtering strategy focused on this effective spectral region to enhance signal stability and reduce noise sensitivity for preliminary model validation. To enhance signal stability and reduce noise sensitivity in this feasibility-stage study, the preprocessing focused on retaining this effective spectral region using a fourth-order Butterworth band-pass filter, together with a 50 Hz notch filter to remove power-line interference. Taking the rectus femoris muscle as an example, Figure 3 compares the raw and filtered sEMG signals and their corresponding power spectra. The filtered signal exhibits significantly reduced noise and clearer activation patterns, suitable for feature extraction.

#### 2.2.2. Feature Extraction

**Figure 4:**
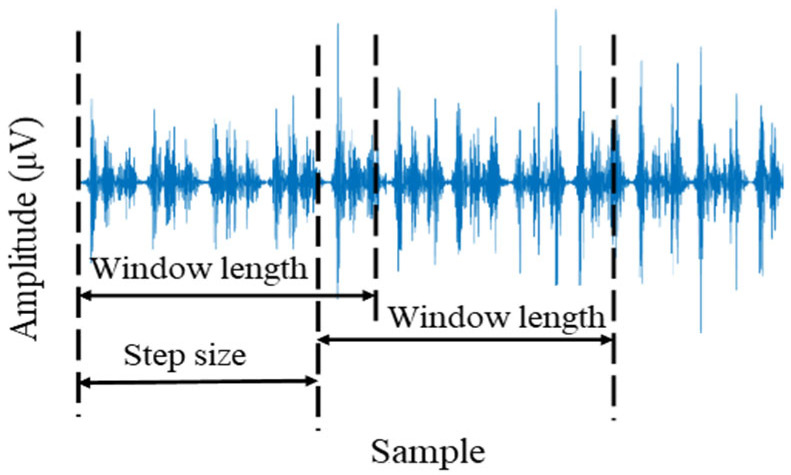
Sliding time-window method.

In this study, regression analysis was conducted to model the relationship between lower-limb joint angles and surface EMG (sEMG) features during level-ground walking. Since both the joint angle and sEMG signals are continuous time series, a window-based segmentation method was employed to extract features suitable for regression modeling. Each continuous signal was divided into equal-length segments using a sliding time window defined by a specified window length and step size. Overlapping windows were applied to improve the temporal resolution of feature extraction [23]. Considering the sampling frequency of 1500 Hz, the window length was set to 150 samples, and the step size to 10 samples, which ensured a balance between signal smoothness and temporal sensitivity. The 100 ms window was adopted for offline analysis to ensure stable feature computation in this feasibility-stage study. The schematic of the sliding time-window method is shown in Figure 4.

**Figure 5:**
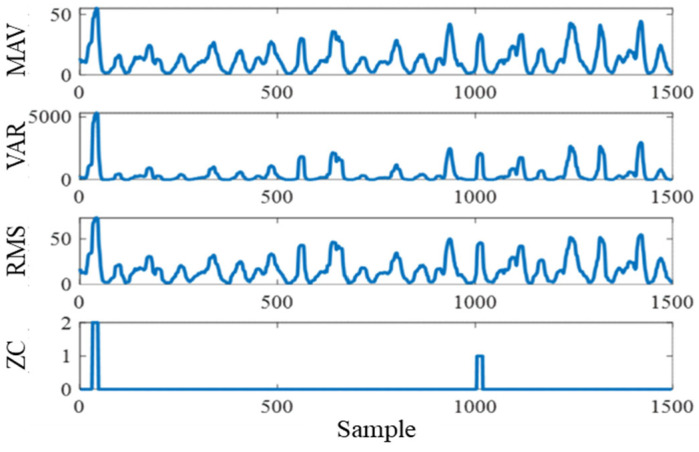
Time-domain features of the rectus femoris sEMG signal.

To capture the temporal characteristics of muscle activity, four commonly used time-domain features—mean absolute value (MAV), variance (VAR), root mean square (RMS), and zero-crossing count (ZC)—were extracted from each sliding window of sEMG data [24]. With five recorded muscles (RF, VL, VM, BF, and ST), a total of twenty candidate features were initially obtained for regression modeling. The extracted MAV, VAR, and RMS features of the rectus femoris EMG signal during walking are shown in Figure 5.

**Figure 6:**
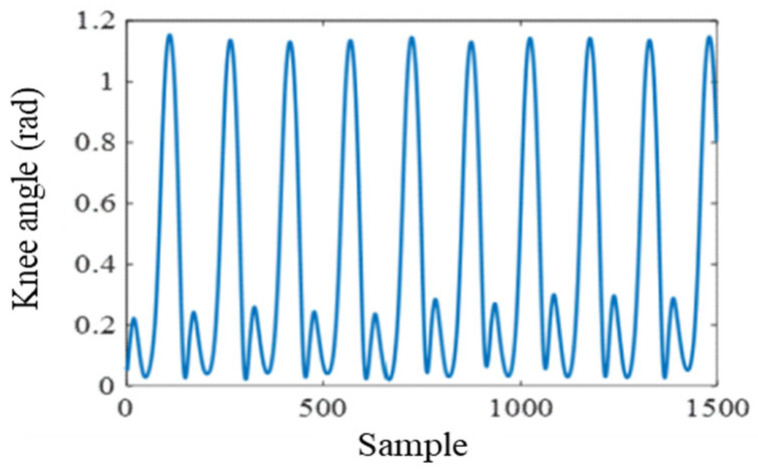
Mean feature extraction of the knee angle signal.

For the kinematic data obtained from the IMU sensors, temporal fluctuations caused by minor limb oscillations may introduce noise. Therefore, the mean value within each time window was used as the representative feature of the knee joint angle. To enable synchronized processing, the 200 Hz kinematic signals were upsampled to 1500 Hz using cubic-spline interpolation, after which both EMG and kinematic series were aligned according to synchronized timestamps. Subsequent windowing and feature computation were performed on the resampled and time-aligned data. The mean feature extracted from the knee angle sequence is shown in Figure 6.

#### 2.2.3. Feature Selection and Correlation Analysis

To improve model prediction accuracy and reduce interference from redundant features, a correlation-based ReliefF optimization method (CORR-ReliefF) was employed for feature screening and dimensionality reduction. This hybrid approach integrates feature-target correlation analysis with sample-distance weighting, ensuring that the selected input features are both representative and effective for regression modeling.

**Figure 7:**
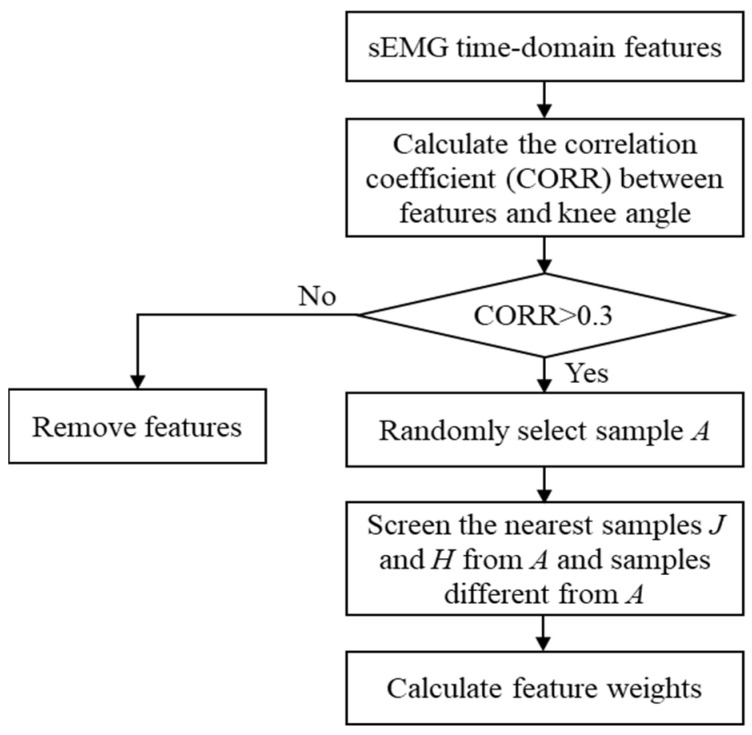
Flowchart of the CORR-ReliefF feature optimization method.

As shown in Figure 7, the optimization process consists of two stages. First, the Pearson correlation coefficient between each candidate feature and the knee joint angle was calculated, and features with |corr| > 0.3 were retained. The correlation threshold of 0.3 was applied as an empirical coarse filter to remove clearly irrelevant features. Given the inherently noisy and non-stationary nature of sEMG signals, correlation values with knee-angle variations tend to be modest even for physiologically meaningful relationships. Therefore, the threshold was intentionally kept low to avoid prematurely discarding potentially informative features. The final feature importance was determined by the subsequent ReliefF weighting rather than by the correlation threshold alone.

Next, the ReliefF algorithm was applied to the retained features. By identifying nearest-neighbor samples within the feature space, the algorithm computed feature weights based on inter-sample distance differences. A higher weight indicates a stronger contribution of that feature to the output variable. This process effectively removes noisy or redundant features while emphasizing those with strong predictive relevance.

**Figure 8:**
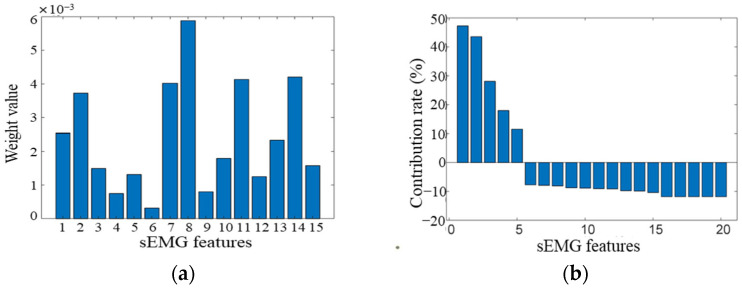
Feature importance evaluation results. (**a**) CORR-enhanced ReliefF feature weights of the 15 retained features; (**b**) Normalized PCA feature contribution ratios of all 20 original features.

Features with low weights—primarily ZC features—were excluded due to weak correlation and limited contribution. After optimization, fifteen high-weight features were selected and used as inputs for the neural network models. The ReliefF feature weights of the fifteen retained features are shown in Figure 8a. The time-domain features of the rectus femoris, vastus lateralis, and semitendinosus muscles exhibited the highest weights, indicating their dominant influence on knee joint motion. Conversely, the ZC features showed weak correlation and were therefore excluded from the final model.

To further validate the reliability of the CORR-ReliefF algorithm, Principal Component Analysis (PCA) was performed to evaluate the contribution of each original feature to the total variance. The feature matrix was first standardized, followed by computation of the eigenvalues and eigenvectors of the feature correlation matrix. The resulting PCA contribution ratios are presented in Figure 8b and summarized in Table 1. The negative values observed in the normalized contribution rates in Figure 8b arise from the centering applied during PCA weight normalization. A negative value reflects a lower relative weight compared with higher-contributing features. The variance features of the semitendinosus, vastus lateralis, rectus femoris, biceps femoris, and vastus medialis muscles exhibited contribution ratios significantly above the mean level. This confirms that the effective information in each sEMG signal was preserved during preprocessing, while the zero-crossing count features showed the lowest contribution, verifying the validity of their exclusion by CORR-ReliefF. The exclusion of ZC features is consistent with prior studies indicating that ZC is more sensitive to noise and less correlated with continuous joint-angle variations [24]. The retained features (mainly MAV, VAR, RMS from RF, VL, and ST muscles) exhibited the strongest relevance to knee-motion dynamics, ensuring more reliable regression mapping for motion-intention prediction.

### 2.3. Motion Intention Mapping Model

#### 2.3.1. BP Neural Network Model

The Back Propagation (BP) neural network is a classical feedforward neural network that updates weights and thresholds through the error backpropagation algorithm to achieve nonlinear mapping between inputs and outputs. In this study, a three-layer BP neural network was constructed, consisting of an input layer, hidden layers, and an output layer. The input comprises the time-windowed and feature-extracted sEMG sequences, while the output corresponds to the knee joint angle at the same time step. The Tanh function was used as the activation function, and the cross-entropy function served as the loss function. Input data were normalized using the Mapminmax method. The detailed network parameters are shown in Table 2.

#### 2.3.2. CNN Model

**Figure 9:**
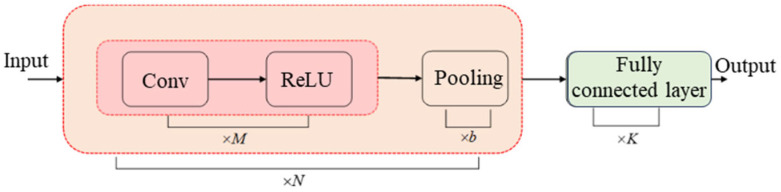
Structure of the CNN model.

The Convolutional Neural Network (CNN) extracts local features from input signals through local receptive fields and weight sharing. In this study, a CNN model was constructed to realize the nonlinear mapping between sEMG features and knee joint angles. The network consisted of a convolutional layer, a pooling layer, and a fully connected layer, as shown in Figure 9. The ReLU function was adopted as the activation function, and the cross-entropy function was used as the loss function. The input data were normalized using the Mapminmax method. The detailed model parameters are listed in Table 3.

#### 2.3.3. LSTM Neural Network Model

**Figure 10:**
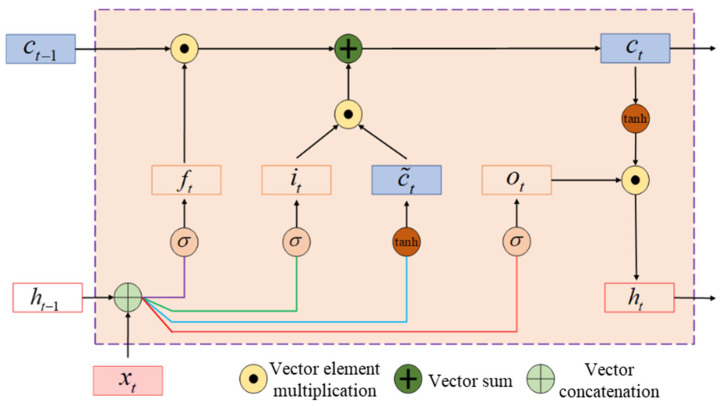
Structure of the LSTM recurrent unit.

The Long Short-Term Memory (LSTM) network is an improved form of the recurrent neural network (RNN). By incorporating forget, input, and output gates, the LSTM network is capable of modeling long-term temporal dependencies, making it suitable for processing time-series data such as gait-related sEMG signals. Figure 10 shows the basic structure of the LSTM neural network. In the figure, $x_{t}$ represents the sEMG feature input at time step t, $h_{t}$ denotes the hidden layer output at time t, and $c_{t}$ is the cell state. The control signals $f_{t}$, $i_{t}$, and $o_{t}$ correspond to the forget gate, input gate, and output gate, respectively, which regulate the transmission and retention of information between time steps. In this study, an LSTM model with one LSTM layer was designed. The learning rate was set to 0.003, the loss function was the cross-entropy function. The model was trained for 80 iterations, and the input data were normalized using the Mapminmax method. Detailed parameters are shown in Table 4.

#### 2.3.4. Model Training and Evaluation Metrics

**Figure 11:**
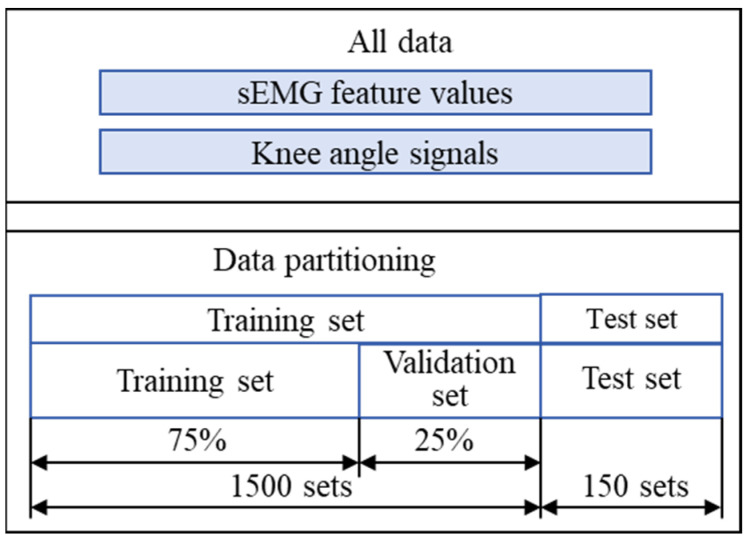
Sample division structure.

The training samples consisted of two parts: feature inputs and labels. The former included the time-domain features of 15 groups of sEMG signals, and the latter corresponded to the knee joint angles. During model training, the dataset was divided into a training set, validation set, and test set. Among them, the training set accounted for 75% and was used for model learning; the validation set accounted for 25% and was used for parameter adjustment and overfitting prevention; an additional 150 independent samples were used as the test set for performance evaluation. The schematic of sample division is shown in Figure 11. Although sliding windows with overlap were used for feature extraction, data partitioning was performed at the level of entire gait cycles to avoid data leakage. Windows extracted from the same gait cycle were assigned exclusively to either the training, validation, or test set, ensuring non-overlap across subsets.

To systematically evaluate the prediction performance of the BP, CNN, and LSTM neural network models, the evaluation indicators were divided into absolute indicators and relative indicators according to the regression characteristics of the models [25].

1. Absolute indicators;

Absolute indicators reflect the magnitude of prediction error. The main indicators include the Mean Absolute Error (MAE), Mean Squared Error (MSE), and Root Mean Squared Error (RMSE), which are calculated as follows:

(1) $$MAE=\frac{1}{N}\sum_{i=1}^{N}\left|y_{i}-{\hat{y}}_{i}\right|$$

(2) $$MSE=\frac{1}{N}\sum_{i=1}^{N}{\left(y_{i}-{\hat{y}}_{i}\right)}^{2}$$

(3) $$RMSE=\sqrt{\frac{1}{N}\sum_{i=1}^{N}{\left(y_{i}-{\hat{y}}_{i}\right)}^{2}}$$

where $y_{i}$ is the actual knee joint angle, ${\hat{y}}_{i}$ is the predicted knee joint angle, and *N* is the total number of samples. The smaller the MAE, MSE, and RMSE values are, the smaller the prediction error, and the higher the accuracy of the model.

2. Relative indicators;

Relative indicators reflect the relationship and fitting degree between the predicted and actual values. The main indicators include the Normalized Mean Squared Error (NMSE), Normalized Root Mean Squared Error (NRMSE), and the Cross-Correlation Coefficient (CORR). Their calculation formulas are as follows:

(4) $$NMSE=\frac{1}{N}\frac{\sum_{i=1}^{N}{\left(y_{i}-{\hat{y}}_{i}\right)}^{2}}{\sum_{i=1}^{N}y_{i}^{2}}$$

(5) $$NRMSE=\frac{\sqrt{\frac{1}{N}\sum_{i=1}^{N}{\left(y_{i}-{\hat{y}}_{i}\right)}^{2}}}{y_{\max}-y_{\min}}$$

(6) $$CORR=1-\frac{\sum_{i=0}^{N-1}{\left(y_{i}-{\hat{y}}_{i}\right)}^{2}}{\sum_{i=0}^{N-1}{\left(y_{i}-{\bar{y}}_{i}\right)}^{2}}$$

Smaller NMSE and NRMSE values indicate lower relative errors, while a CORR value closer to 1 indicates stronger correlation between the predicted and actual results, reflecting better fitting performance of the model.

### 2.4. Direct Myoelectric Control Based on the Human-Machine Coupling Model

To realize adaptive control of an intelligent prosthetic knee joint, a direct myoelectric control system based on the human-machine coupling model was constructed in this study, building upon the previously established motion intention recognition model. The proposed method uses the knee joint angle signal predicted by the LSTM neural network as the input to the human-machine coupling control module, thereby enabling real-time adjustment of the hydraulic damping of the prosthetic knee joint. This allows the prosthesis to dynamically synchronize its movement with the wearer’s motion intention.

#### 2.4.1. Overall Structure of the Control System

**Figure 12:**
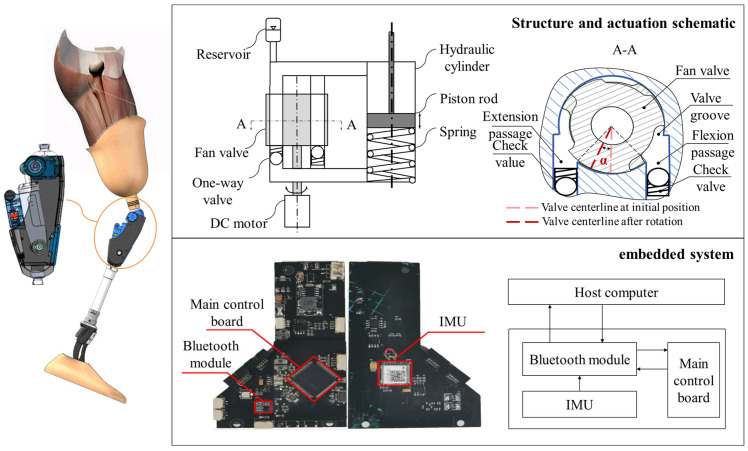
Prototype of the variable-damping hydraulic knee prosthesis.

The experimental prosthesis used in this study was a self-designed variable-damping hydraulic knee prosthesis [26,27]. The prosthesis adopts a dual-chamber hydraulic cylinder structure, in which a servo motor-driven rotary valve regulates the flow rate to control the magnitude of hydraulic damping. The system features a compact design and can automatically adjust damping across different gait phases to achieve compliant motion control. The prototype of the prosthesis is shown in Figure 12.

**Figure 13:**
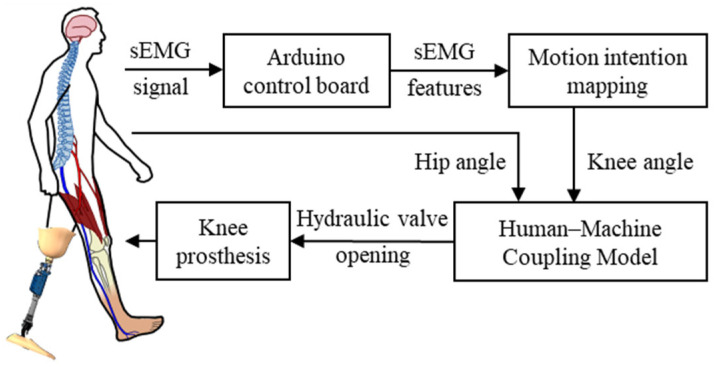
The direct myoelectric control system based on the human-machine coupling model.

The control system consists of three main components: (1) sEMG acquisition and motion intention mapping module: this module uses the EMG acquisition system to collect real-time sEMG signals from the major lower-limb muscles. The LSTM neural network model is then employed to map the sEMG signals to the knee angle; (2) Human-machine coupling control module: based on the predicted knee joint angle, this module computes the opening of the hydraulic valve, which is adjusted by a servo motor to regulate hydraulic damping and realize torque self-adaptation; (3) Prosthetic actuation module: this module includes the hydraulic knee prosthesis, controller, motor driver, and sensor units, which execute flexion-extension motions and provide real-time feedback of motion states. The overall block diagram of the control system is shown in Figure 13.

#### 2.4.2. Human-Machine Coupling Model

The human-machine coupling model describes the interactive dynamics between the motion intention of the human lower limb and the mechanical response of the prosthesis. The model integrates the biomechanical characteristics of the human knee joint with the damping properties of the hydraulic system, converting the predicted knee angle signal from the neural network into a control signal for the hydraulic valve.

The model was developed based on the authors’ previous research [27]. A simplified double-pendulum model was used to represent the lower-limb dynamics, and the energy conservation principle of the hydraulic system was applied to establish the damping regulation equation. This model realizes a continuous mapping from the predicted knee angle to the hydraulic valve opening, allowing the system to automatically adjust damping according to gait variations, thereby achieving smooth and natural prosthetic flexion and extension. A detailed derivation and mathematical formulation of this coupling model can be found in [27].

Through parameter identification and coupled solution, the nonlinear relationship between the valve opening, knee angle, and angular velocity can be expressed as:

(7) $$A=f(\theta ,\dot{\theta })$$

where *A* represents the hydraulic valve opening, θ is the knee joint angle, and $\dot{\theta }$ is the angular velocity. This relationship enables the system to adaptively regulate damping characteristics according to the real-time motion state of the human body.

#### 2.4.3. Control Implementation and Wearing Experiment

**Figure 14:**
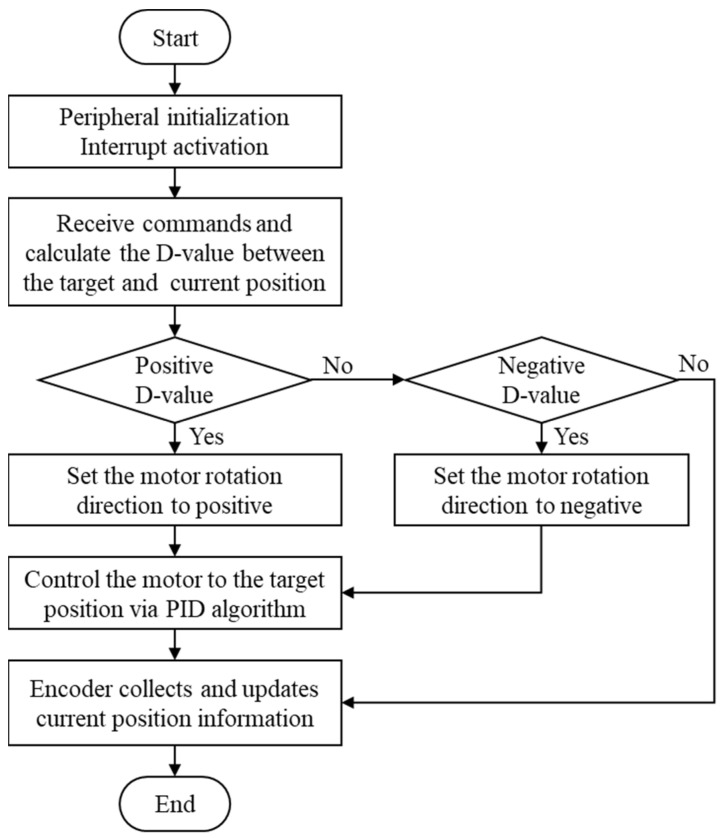
Closed-loop control flow of the servo motor.

Under the human-machine coupling control framework, the knee joint angle signal output by the LSTM model is converted by the coupling model into a hydraulic valve opening command. The servo motor then adjusts the valve spool position in real time to achieve continuously variable damping control. The closed-loop control process of the servo motor is shown in Figure 14.

**Figure 15:**
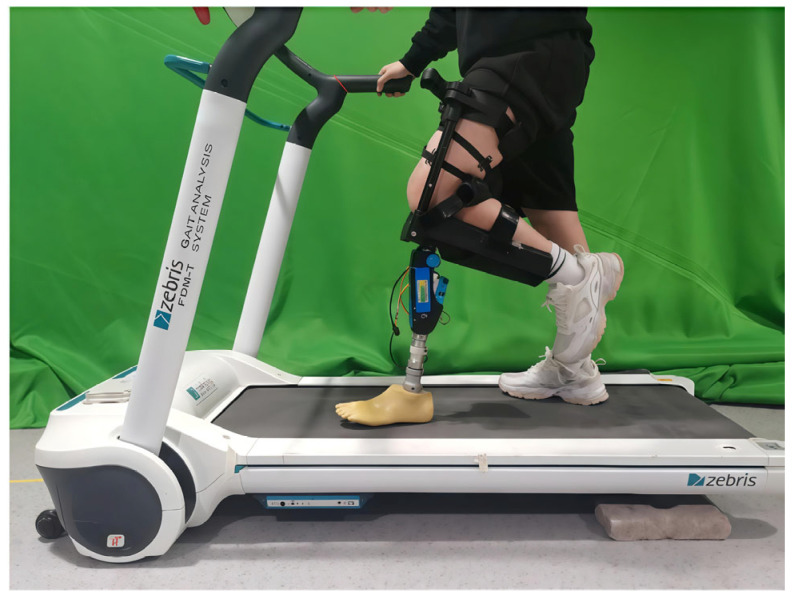
Experimental setup for prosthesis wearing test.

To verify the feasibility of the control system, an experimental platform integrating EMG acquisition, neural network prediction, and hydraulic control was established. As shown in Figure 15, an L-shaped test socket was used to allow a healthy participant to simulate the residual-limb-socket interface of a transfemoral amputee. The participant’s thigh was positioned inside the socket in a flexed posture to ensure a secure and safe mechanical connection with the prosthetic knee. This apparent flexed configuration reflects only the supporting structure of the test socket and the instantaneous moment captured in the photograph. During walking, the prosthetic knee was not maintained at a fixed angle; rather, it continuously adjusted its flexion-extension motion according to the real-time EMG-based motion-intention mapping and the human-machine coupling control strategy.

## 3. Results

### 3.1. Motion Intention Mapping Results

**Figure 16:**
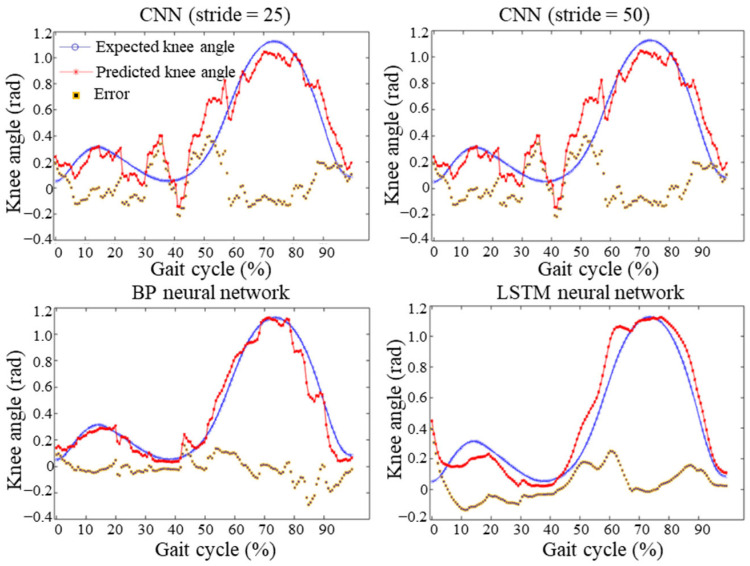
Prediction results of the four neural network models.

**Figure 17:**
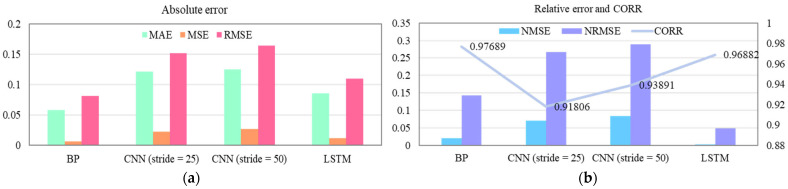
Comparison of error evaluation results among different neural network models. (**a**) Absolute error indicators; (**b**) Relative error and correlation coefficient indicators.

The NRMSE and CORR evaluation results of the four neural network models—BP, CNN (stride = 25), CNN (stride = 50), and LSTM—are summarized in Table 5, while their corresponding prediction results are illustrated in Figure 16. In these plots, the blue curve represents the actual knee joint angle data, the red curve denotes the predicted knee joint angle data, and the yellow-purple curve shows the prediction error between them. The absolute evaluation indicators (MAE, MSE, and RMSE) for the four models are presented in Figure 17a, whereas the relative evaluation indicators (NRMSE, NMSE, and CORR) are depicted in Figure 17b.

As shown in Figure 16 and Figure 17 and Table 5, both the BP neural network and the LSTM model demonstrated significantly superior performance in terms of absolute error, relative error, and correlation coefficient compared to the CNN models. Specifically, the BP neural network achieved the highest correlation coefficient (0.97689), while the LSTM model obtained the lowest normalized root mean square error (4.8167%). It is noteworthy that both models achieved correlation coefficients greater than 0.95 and NRMSE values lower than 15%, meeting the regression model feasibility criteria. These results indicate that the BP neural network and LSTM models are well suited for predicting human lower-limb motion intention based on sEMG signals.

**Figure 18:**
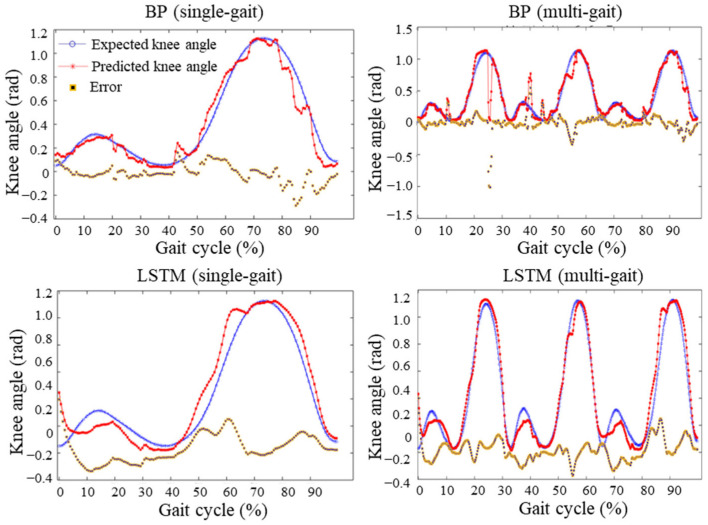
Prediction results of the models under single-gait and multi-gait test sets.

**Figure 19:**
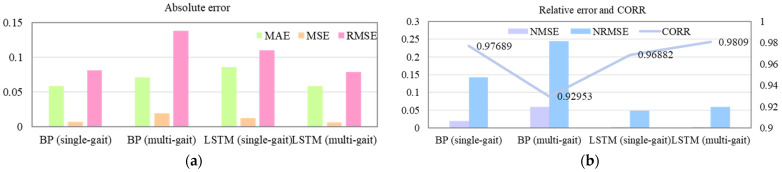
Comparison of model performance under single-gait and multi-gait test sets. (**a**) Absolute error comparison; (**b**) Relative error and correlation coefficient comparison.

According to the results of the 150 data groups, corresponding to one gait cycle in the test set, both the BP neural network and LSTM models satisfied the regression feasibility requirements. Considering the hardware response speed and safety of the intelligent prosthetic knee joint, further validation was performed using three consecutive gait cycles as the test dataset to evaluate the prediction performance of both models over multiple gait periods. The results are shown in Figure 18, where the blue curve represents the actual knee joint angle, the red curve represents the predicted angle, and the yellow-purple curve indicates the prediction error. The results of the BP and LSTM models under both single- and multi-gait test sets are presented in Table 6. The corresponding absolute evaluation indicators (MAE, MSE, RMSE) are illustrated in Figure 19a, while the relative indicators (NRMSE, NMSE, and CORR) are depicted in Figure 19b.

**Figure 20:**
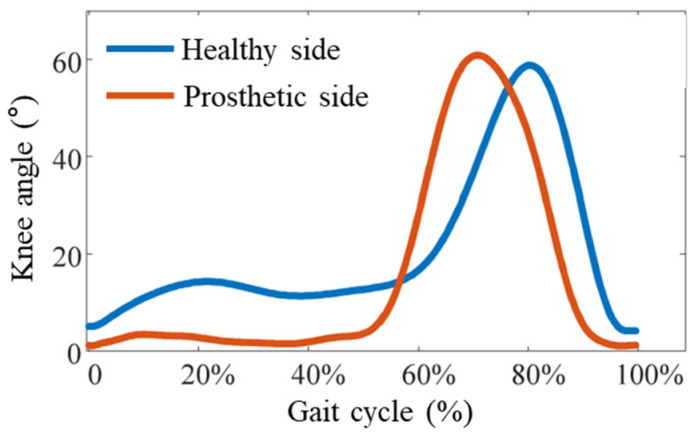
Experimental results of the prosthesis-wearing test.

By comparing the results of the single- and multi-gait test sets, it can be seen from Figure 19 and Figure 20 and Table 6 that the BP neural network exhibited a significant increase in prediction error for multi-gait data, with the NRMSE rising to 24.409% and the correlation coefficient decreasing to 0.92953. In contrast, the LSTM model showed only a slight increase in NRMSE to 5.8893%, while its correlation coefficient improved to 0.9809. Therefore, for multi-gait knee joint angle prediction, the BP neural network no longer meets the regression feasibility criteria.

Comprehensive analysis of the above results shows that the LSTM model is the optimal approach for motion intention mapping aimed at direct myoelectric control, as it can accurately predict human lower-limb motion intention based on sEMG signal features. This superiority is closely related to the inherent characteristics of LSTM, whose long-term and short-term memory mechanisms make it particularly suitable for modeling time-dependent sequences. Consequently, the LSTM network provides more reliable regression mapping between sEMG signals and knee angles.

### 3.2. Direct Myoelectric Control Results

After averaging the bilateral knee joint angle signals collected within one minute, the experimental results were analyzed. As shown in Figure 20, the gait pattern of a transfemoral amputee differs from that of a healthy individual. Due to the pressure of the prosthetic socket on the residual limb and the user’s individual adaptation habits, the knee angle of the healthy side shows slight deviation from normal. Nevertheless, during the stance phase, the healthy knee still exhibits flexion, with a maximum flexion angle of approximately 16°, and reaches about 60° during the swing phase. By analyzing the knee angle on the prosthetic side, it can be observed that, for safety considerations, the damping of the prosthetic knee was maximized during the stance phase to ensure stability; therefore, the knee angle remained close to 0° in this phase. During the swing phase, the prosthetic knee performed normal flexion and extension, achieving a maximum flexion angle of about 60°.

To objectively evaluate the experimental results and quantify the gait symmetry between both lower limbs, this study adopted two common metrics: the Symmetry Index (SI) and the Ratio Index (RI). Their calculation formulas are as follows:

(8) $$SI=\left|\frac{2\left(X_{H}-X_{D}\right)}{X_{H}+X_{D}}\right|\times 100\%$$

(9) $$RI=\left|1-\frac{X_{D}}{X_{H}}\right|\times 100\%$$

where $X_{H}$ represents the gait time of the healthy limb and $X_{D}$ represents that of the prosthetic limb. The closer the SI and RI values are to zero, the better the gait symmetry of the lower limbs. When these indices are less than 10%, the gait is generally considered to meet the symmetry requirements. The SI and RI quantify left-right similarity of knee-angle waveforms within a gait cycle and are therefore minimally influenced by gait speed, stride length, or cycle-to-cycle variability. The quantitative analysis results of the prosthesis-wearing experiment are shown in Table 7.

As shown in Table 7, the lower-limb motion exhibited good gait symmetry throughout the entire gait cycle, the stance phase, and the swing phase, all satisfying the symmetry criteria (SI, RI < 10%). This demonstrates that the proposed direct myoelectric control method meets the control requirements for stable prosthetic movement. Through analysis and summary of the prosthesis-wearing experimental results, it can be concluded that the direct myoelectric control strategy proposed in this study enables the prosthetic knee joint to produce smooth, human-like gait motion. This verifies the feasibility and effectiveness of the method proposed in this research.

## 4. Discussion

Based on experiments conducted on healthy subjects during level-ground walking, this study proposed a lower-limb motion intention recognition method using a Long Short-Term Memory (LSTM) neural network, which was further integrated with a human-machine coupling model to achieve direct myoelectric control of a variable-damping hydraulic knee prosthesis. Experimental results demonstrated that the LSTM model achieved high prediction accuracy and stability in the regression analysis of lower-limb sEMG signals. The predicted knee joint angle curve closely matched the actual measured data, with a Normalized Root Mean Square Error (NRMSE) of 5.89% and a Correlation Coefficient (CORR) of 0.981, which were significantly better than those of the BP and CNN models. These results indicate that the LSTM model can effectively capture the temporal dependencies of sEMG signals, enabling smooth and continuous prediction of knee joint motion. The LSTM architecture was adopted in this feasibility-stage study due to its stability, suitability for small datasets, and its extensive use as a baseline method in EMG-driven regression research [28,29]. Future work will involve larger multi-participant datasets, including amputee users, enabling the comparison of more advanced neural network architectures as well as comprehensive statistical evaluation through variability analysis, confidence intervals, and significance testing.

Compared with traditional proportional myoelectric control methods, the proposed model does not rely on explicit linear mapping relationships. Instead, it automatically learns the nonlinear correspondence between muscle activity and kinematic features through deep network structures, better reflecting the dynamic coupling between muscle activation and joint motion. Compared with the feedforward neural network model developed by Coker et al. [15] and the hybrid models proposed by Li et al. [16] and Sun et al. [17], this study integrates human-machine coupling control, achieving coordinated linkage between neural network predictions and the hydraulic actuation mechanism, thereby producing smoother and more natural prosthetic motion responses.

In the human-machine coupling control experiments, the flexion-extension trajectory of the prosthetic knee closely followed that of the healthy limb, exhibiting stable gait with no noticeable delay or oscillation. The gait symmetry indices, SI and RI, were both below 10%, confirming that the system successfully achieved coordinated and biomimetic gait during dynamic walking.

Compared with traditional open-loop proportional control prostheses, the proposed direct myoelectric control method exhibited improved damping adaptability and output continuity across multiple gait cycles, validating the feasibility of integrating deep learning-based intention recognition with a hydraulic control system.

Another important physiological factor influencing EMG-kinematics mapping is antagonist co-contraction. Variations in flexor-extensor co-activation can modulate limb stiffness and alter the amplitude and distribution of EMG signals, thereby affecting knee-angle estimation accuracy [30]. Although co-contraction levels remained relatively stable during controlled treadmill walking, greater variability may occur under perturbed gait, fatigue, or in amputee users with altered neuromuscular strategies. Future work will consider incorporating co-contraction-related features (e.g., activation ratios between antagonistic muscle groups) or multimodal sensing to enhance the robustness of the estimation model. While this study focuses on lower-limb prosthetic control, the proposed EMG-based intention mapping framework could potentially be adapted for upper-limb prostheses with appropriate task-specific modifications.

Nevertheless, several limitations remain. First, this study was conducted using data from a single healthy participant, which limits generalizability and does not capture inter-subject variability in sEMG signals, such as differences related to adipose-tissue thickness, electrode-placement variation [18], or joint-angle-dependent modulation during locomotion [19]. Second, the recorded muscle groups reflect intact-limb activation patterns. Residual-limb muscle availability and activation characteristics in transfemoral amputees may differ depending on amputation level and anatomical condition, suggesting that appropriate adaptation—such as individualized EMG-channel selection or alternative residual-limb muscle sites—may be required when applying the method to amputee users. In addition, the neural network model was trained offline without subject-specific or online adaptive adjustment. As an early-stage feasibility verification, this work primarily aims to establish the foundational validity of the LSTM-based motion-intention mapping and the proposed human-machine coupling control scheme.

Future work will expand data collection to a larger and more diverse cohort, including amputee participants, and incorporate real-time processing, online learning, and multimodal sensing (e.g., IMUs, plantar pressure) to enhance robustness under varying gait conditions. The EMG sensor set will also be extended to include additional muscle groups, particularly for amputee users who may rely more heavily on hip compensation strategies. Ultimately, clinical trials with amputee subjects will be conducted to evaluate the practicality and applicability of the proposed direct myoelectric control strategy.

## 5. Conclusions

This study proposed a direct myoelectric control method based on a Long Short-Term Memory (LSTM) neural network for human motion intention recognition, which was further integrated with a human-machine coupling dynamic model to realize biomimetic control of a variable-damping hydraulic knee prosthesis. The research results demonstrate that the LSTM model can effectively extract the temporal features of sEMG signals and achieve high-precision regression prediction of knee joint angles, with prediction accuracy and stability superior to those of the BP and CNN models. By integrating the neural network output with the hydraulic control system, a continuous mapping from sEMG signals to prosthetic flexion-extension motion was established. The prosthetic gait exhibited smooth and symmetric motion, verifying the feasibility of the proposed method in achieving coordinated human-machine control. This work provides a novel approach for the direct myoelectric control of intelligent prostheses. In future work, experiments will be conducted with amputee participants to further evaluate the model’s adaptive capability under different gait patterns and terrain conditions. Additionally, by incorporating multimodal sensing and real-time learning algorithms, the robustness and real-time responsiveness of the control system will be further enhanced.

## Figures and Tables

**Table 1:** Features with contribution ratios above the mean level.

Feature	Contribution (%)
Semitendinosus (Variance)	47.28
Vastus Lateralis (Variance)	43.57
Rectus Femoris (Variance)	28.09
Biceps Femoris (Variance)	17.97
Vastus Medialis (Variance)	11.57

**Table 2:** Parameters of the BP neural network model.

Parameter	Value
Number of input nodes	1
Number of hidden layers	7
Number of neurons per hidden layer	93
Activation function	Tanh
Loss function	Cross-entropy
Number of iterations	800
Normalization method	Mapminmax

**Table 3:** Parameters of the CNN model.

Parameter	Value
Number of input nodes	Windows × 5
Number of output nodes	1
Number of fully connected layers	1
Neurons per fully connected layer	7
Number of convolutional layers	1
Convolution kernel size	3 × 3
Number of pooling layers	1
Pooling function	Average pooling
Activation function	ReLU
Gradient threshold	0.0001
Loss function	Cross-entropy
Number of iterations	800
Normalization method	Mapminmax

**Table 4:** Parameters of the LSTM model.

Parameter	Value
Number of output nodes	1
Number of hidden layers	1
Number of iterations	80
Learning rate	0.003
Gradient threshold	0.0001
Loss function	Cross-entropy
Normalization method	Mapminmax

**Table 5:** NRMSE and CORR results.

Model	CORR	NRMSE
CNN (stride = 25)	0.91806	26.654%
CNN (stride = 50)	0.93891	28.906%
BP Neural Network	0.97689	14.287%
LSTM	0.96882	4.8167%

**Table 6:** Results under single-gait and multi-gait test sets.

Dataset	Model	CORR	NRMSE
Single gait	BP Neural Network	0.91806	26.654%
	LSTM	0.93891	28.906%
Multi-gait	BP Neural Network	0.97689	14.287%
	LSTM	0.96882	4.8167%

**Table 7:** Quantitative analysis results of the prosthesis-wearing experiment.

Evaluation Metric	Value
Complete Gait Cycle (SI)	6.67%
Stance Phase (RI)	6.58%
Swing Phase (RI)	6.54%

## Data Availability

The data supporting this study’s findings are available upon reasonable request from the authors.
